# Internet-Based Interventions for the Prevention and Treatment of Mental Disorders in Latin America: A Scoping Review

**DOI:** 10.3389/fpsyt.2019.00664

**Published:** 2019-09-13

**Authors:** Álvaro Jiménez-Molina, Pamela Franco, Vania Martínez, Pablo Martínez, Graciela Rojas, Ricardo Araya

**Affiliations:** ^1^Millennium Nucleus to Improve the Mental Health of Adolescents and Youths (Imhay), Santiago, Chile; ^2^Millennium Institute for Research in Depression and Personality (MIDAP), Santiago, Chile; ^3^Millennium Nucleus in Social Development (DESOC), Santiago, Chile; ^4^Programa de Doctorado en Psicoterapia, Facultad de Ciencias Sociales and Facultad de Medicina, Pontificia Universidad Católica de Chile and Universidad de Chile, Santiago, Chile; ^5^CEMERA, Facultad de Medicina, Universidad de Chile, Santiago, Chile; ^6^Escuela de Psicología, Facultad de Humanidades, Universidad de Santiago de Chile, Santiago, Chile; ^7^Hospital Clínico, Universidad de Chile, Santiago, Chile; ^8^Centre for Global Mental Health, Institute of Psychiatry, Psychology & Neuroscience, King’s College London, London, United Kingdom

**Keywords:** Internet, technology, telepsychiatry, mental disorders, prevention, treatment, Latin America

## Abstract

**Background:** There is a huge gap in the treatment of mental disorders in Latin America, especially among socioeconomically disadvantaged groups. Given the sharp increase in Internet access and the rapid penetration of smartphones in the region, the use of Internet-based technologies might potentially contribute to overcoming this gap and to provide more widely distributed and low-cost mental health care in a variety of contexts.

**Methods:** We conducted a scoping review of the literature in order to systematically map the existing evidence on use of Internet-based interventions for prevention, treatment, and management of mental disorders across Latin American countries, as well as to identify existing gaps in knowledge. Six electronic databases were searched for published papers (PubMed, Embase, CINAHL, Web of Science, SciELO, and CENTRAL).

**Results:** After the eligibility assessment, we identified 22 Internet-based studies carried out in Latin America for prevention, treatment, education, or facilitating self-management of mental disorders. Included studies mainly targeted depression (*n* = 11), substance misuse (*n* = 6), anxiety (*n* = 3), and mental health literacy for education and health professionals (*n* = 2). Most studies were undertaken in Brazil (*n* = 6), Mexico (*n* = 5), and Chile (*n* = 4). Only 3 studies were randomized controlled trials (RCTs), 4 were pilot RCTs, and 15 were naturalistic, acceptability, or feasibility studies. The three RCTs identified showed disparate results, but overall, there are challenges to face. Better results are seen in the short-term (postintervention or after 3 months), but most studies do not explore outcomes for long enough (follow-up after 6 or 12 months). Most of the feasibility and pilot studies showed reasonably good acceptability for a wide range of strategies but difficulties to engage and retain participants for long enough or adhering to established protocols.

**Conclusion:** This study shows that Internet-based interventions for the prevention and treatment of mental disorders are growing rapidly in Latin America, but there are few studies on effectiveness and cost effectiveness, making it difficult to provide the evidence needed to justify scaling up these interventions.

## Introduction

Mental disorders are common in Latin America. For instance, depression is the largest cause of disability in the region, and mental health problems represent one-third of all years lost for disability in Latin American countries (1). A striking imbalance exists between government spending on mental health and the related disease burden (2), and the treatment gap for mental disorders in Latin America is huge with ∼80% of those suffering from depression receiving no help at all (1). This is even more marked among socioeconomically disadvantaged groups (3, 4).

Among many reasons for this situation, the lack of trained personnel figures prominently. To address this problem, the strategy of task shifting/sharing has been implemented, in which nonmedical health workers deliver most, if not all, of the treatment for mental disorders (5, 6). A model developed in Latin America became the first task-shifted treatment program to show its cost effectiveness from low-middle income countries (LMICs) (7, 8), and it was subsequently successfully adapted and replicated in other LMICs (9, 10). Although this is a promising strategy, it has become increasingly clear that nonmedical health workers have an increasing number of competing demands, and other barriers, such as access to health care clinics and stigma, still pose major hurdles to improve coverage in the region and elsewhere. Expanding task shifting to include the use of technology to either complement or even replace health workers might potentially contribute to overcoming some of these obstacles and reducing the treatment gap.

There has been a rapid growth in the development of Internet-based interventions for preventing and treating mental health problems in developed countries (11). For instance, approximately one-third of all health-related apps are on mental health topics (12). Nonetheless, there are still a number of challenges to be faced by this rapidly growing industry, in particular those related to the evaluation of these products and interventions affecting countries at all levels of development (12, 13).

Given the sharp increase in Internet access and the rapid penetration of smartphones in Latin America and elsewhere (12), the integration of Internet-based interventions may offer a potential to provide more widely distributed and low-cost mental health care (12–14). Users have the opportunity to access help, often based on proven effective clinical guidelines, at a convenient time and wherever they are, saving precious time and unnecessary transport costs. Internet-based programs and digital technologies have also the potential to be important tools for disseminating sustainable training programs and to support and supervise mental health workers (11, 14). In spite of all these merits, there has been limited use of digital advances in mental health in LMICs, and some questions remain about their effectiveness in less-developed countries and settings with restrained resources (11). Previous reviews have found virtually no studies on Internet-based solutions in Latin America that can be scaled up in real health-care contexts (15–17).

We conducted a scoping review of the literature in order to systematically map the existing evidence on use of Internet-based interventions for mental disorders in Latin America, as well as to identify existing gaps in knowledge. The following research question was formulated: What is known from the literature about Internet-based interventions for the prevention, treatment, or management of mental disorders across Latin American countries? We consider how Internet-based interventions could overcome barriers to improving access to mental health care in these countries, and we evaluate the main strengths and limitations of the interventions developed in the region.

## Method

We carried out a systematic search of the literature on Internet-based studies in mental health undertaken in Latin America. While our initial intention was to do a systematic review, we decided to do a scoping review because potentially eligible studies used diverse study designs (many studies were pilot, acceptability, or feasibility studies), recruited diverse population groups with a wide range of mental disorders, and reported heterogeneous outcomes. We use the PRISMA Extension for Scoping Reviews (PRISMA-ScR) to conduct the review and report the results (18). A systematic review would have required greater consideration of intervention effectiveness (19), whereas scoping reviews are useful for answering much broader questions. Likewise, a scoping review allowed for greater discussion of important areas in which we believe that Internet and digital technologies could yield considerable gains towards addressing mental disorders in Latin America.

### Eligibility Criteria

We searched for published papers in peer-reviewed journals, written in English or Spanish, indexed from inception to April 5, 2019. The following study eligibility criteria were applied, according to the Population, Intervention, Comparison, Outcomes, and Study design (PICOS) framework (19):

(P) Individuals living in Latin America, without distinction of age, sex, or ethnicity. The scope of this review allowed for the inclusion of subjects experiencing varying degrees of psychopathology, determined either through validated self-reported scales, observer-rated questionnaires, or clinical interviews. For instance, in the case of interventions aimed at the promotion of mental health and/or prevention of mental disorders, individuals may have been identified as healthy or at-risk of mental ill health. We did not exclude any type of mental health problem, as such, we included common mental disorders/symptoms (e.g., depression and/or anxiety), serious mental illnesses (e.g., schizophrenia or bipolar disorder), and alcohol and other substance use disorders. Moreover, we also considered for inclusion those studies in which participants were mental health care providers, whether they were lay health care-workers, primary health-care personnel, or specialized mental health-care professionals.

(I) Internet-based interventions aimed at the promotion of mental health and the prevention and/or treatment of mental disorders, targeted at healthy, at-risk, or mentally ill individuals. Additionally, we included supporting and/or training Internet-based interventions for mental health-care providers. For purposes of this scoping review, interventions must have been based on or supported by Internet and digital technologies (i.e., web and mobile based), with varying degrees of human support. Importantly, these interventions may have been provided alongside other treatment components, regardless of their technological status (e.g., as in the case of blended interventions, which combines face-to-face and Internet-based approaches).

(C) Any intervention that is not based on Internet or digital technologies was considered as a comparator or control. However, studies without comparison/control groups were also considered.

(O) To be included, studies must have used validated self-reported scales, observer-rated questionnaires, or clinical interviews and must have provided postintervention assessments or follow-up evaluations. We do not consider specific restrictions regarding the length of follow-up periods. Outcomes considered included measures of positive mental health, mental disorders or symptoms, information or attitudes (e.g., knowledge) related to mental illness, and use, adherence, or satisfaction with health services.

(S) We did not exclude studies by their design; rather, the rationale for the inclusion of studies was based on studies’ aims. Thus, we identified two study types of interest to our scoping review: feasibility studies, dealing with issues such as the acceptability, demand, and practicality of interventions (i.e., “Can it work?”); and efficacy/effectiveness studies (which addresses the question: “Does the intervention work?”). This meant that we expected to include quantitative, qualitative, and mixed methods studies in order to consider different aspects of Internet-based interventions.

Papers were excluded if they did not fit into the conceptual framework of the study.

### Information Sources and Search

To identify potentially relevant documents, the following bibliographic databases were searched: PubMed, Embase, CINAHL, Web of Science, SciELO, and CENTRAL. The appendix shows the complete search strategy used. The search results were exported into EndNote, and duplicates were removed. In addition, we searched reference lists of included studies and other systematic reviews.

Included studies evaluated Internet-based interventions targeting depression, including symptoms and disorders; serious mental illnesses, including schizophrenia or bipolar disorder; alcohol and other substance-misuse disorders; and other common mental health conditions, including anxiety disorders and posttraumatic stress disorder. The included studies considered intervention involving the use of Internet and digital technologies, such as telepsychiatry, web-based, wearable devices, and mobile applications.

We excluded discussion articles, program descriptions, study protocols, and conference abstracts.

### Selection of Sources of Evidence

To increase consistency among reviewers, three reviewers (ÁJ-M, PF, and PM) carried out study selection and data collection in an independent manner. These three reviewers evaluated the titles, abstracts, and then full text of all publications identified. The study selection process was documented using the PRISMA-ScR flow diagram (18).

Data were extracted and synthetized for country of origin, sample description, study design, intervention description, and primary outcome. Three authors (RA, VM, and GR) reviewed the list of included studies and the data included in the summary tables to confirm accuracy in data extraction. All authors reviewed the final tables.

### Risk of Bias Across Studies

A key difference between systematic and scoping reviews is that the latest are generally conducted to provide an overview of the existing evidence regardless of methodological quality or risk of bias. However, we performed a risk of bias assessment of included randomized controlled trials (RCTs) according to the criteria of the Cochrane Collaboration tool for assessing risk of bias (19). Two reviewers (ÁJ-M and PF) carried out risk of bias assessment in an independent manner.

### Synthesis of Results

We grouped the studies according to the type of design: randomized controlled trials, pilot randomized controlled trials, and naturalistic, acceptability or feasibility studies. Narrative techniques were the selected approach for data analysis and synthesis, with due emphasis on study characteristics and risk of bias assessment to interpret study results. We also use comparative tables to facilitate the analysis of study characteristics and results.

## Results

**Figure 1:**
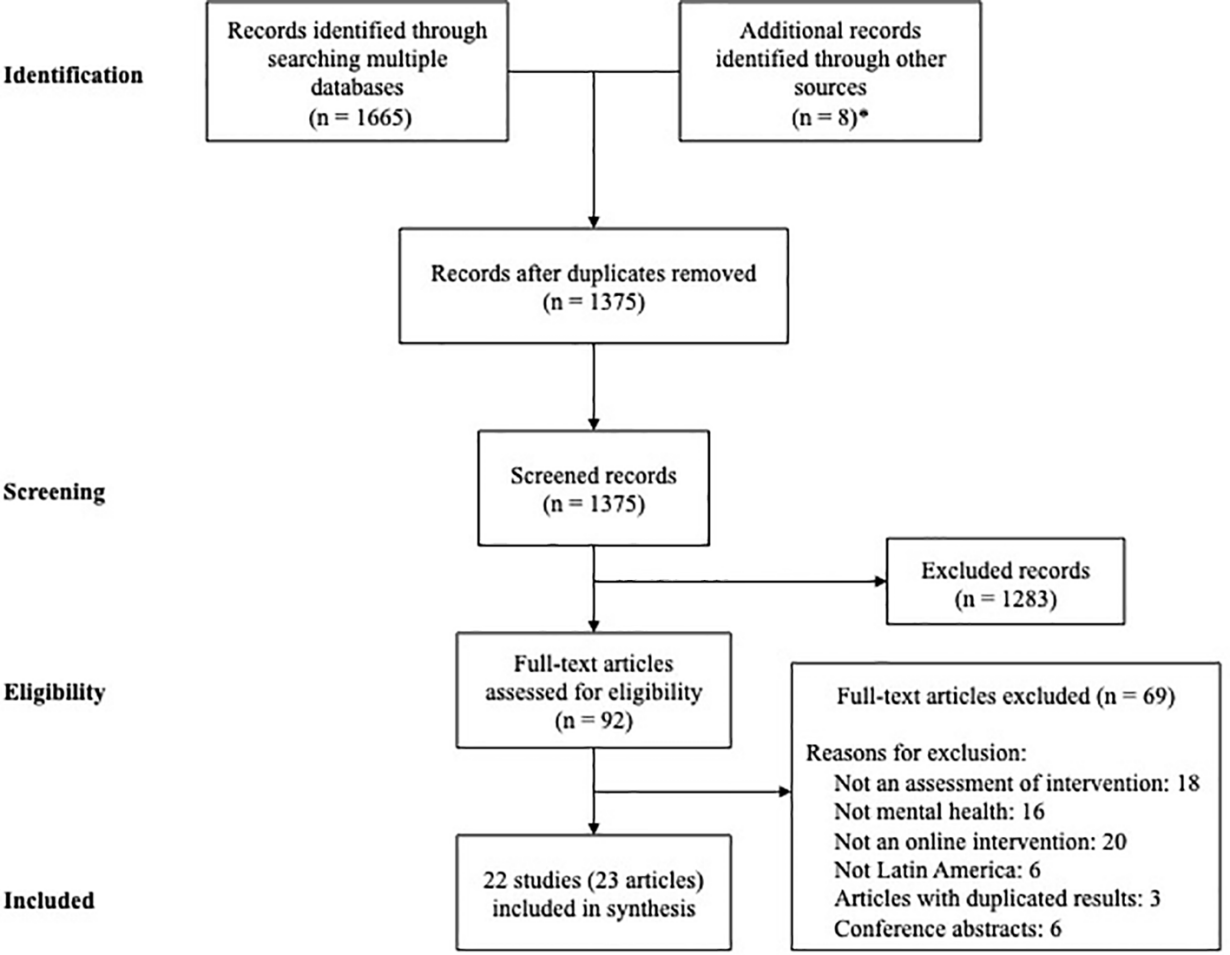
PRISMA-ScR Flow diagram. *Seven articles reported in other review + one article published by one of the authors.

After duplicates were removed, a total of 1,375 records were screened. Based on the title and the abstract, 1,283 were excluded, with 92 full text to be retrieved and assessed for eligibility. Of these, 69 were excluded for the following reasons: 18 did not directly evaluate an intervention, 16 did not specifically address mental health issues, 20 were not Internet-based interventions, 6 were not implemented in Latin America, and 3 reported duplicate results in other publications. We also excluded six studies because they were reported in conference abstracts. After the eligibility assessment, we identified 22 Internet-based studies carried out in Latin American countries for the prevention, treatment, education, or facilitating self-management of mental disorders considered eligible for this review (see **Figure 1**).

### Characteristics of Sources of Evidence

Most included studies were undertaken in Brazil (*n* = 6), Mexico (*n* = 5), and Chile (*n* = 4). We found four multisite studies conducted in several countries in South and Central America. Included studies mainly targeted depression (*n* = 11), substance misuse (*n* = 6), anxiety (*n* = 3), and mental health literacy for education and health professionals (*n* = 2).

The studies included in this review were classified and grouped according to the research design (RCTs, pilot RCTs, and other studies) and the type of intervention: telepsychiatry/psychology, guided Internet-based self-help programs, unguided Internet-based self-help programs, and Internet-based programs for education and training of health/education workers.

Telepsychiatry/psychology interventions reported here use the Internet to allow simultaneous and/or time-delayed communication between a patient or primary health-care professional and a mental health specialist (psychiatrist/psychologist) *via* video conferencing, chat, or other electronic platform. These interventions can enable mental health specialists to provide remote services or supervise nonspecialized care teams. Guided Internet-based self-help programs were automated interventions that included some kind of human support but considerably less than in face-to-face therapy. Unguided Internet-based self-help programs were automated interventions with no human interaction at all. Finally, Internet-based programs for education and training of health/education workers were interventions using Internet to access mental health knowledge (e.g., e-learning) to professionals who may have a role in preventing, diagnosing, or managing mental disorders.

We identified three RCTs testing the effectiveness of Internet-based interventions: a web-based mental health training program for school teachers (20), an unguided web-based alcohol intervention (21, 22), and a remote collaborative care program (23). Four included studies were pilot RCTs. One evaluated the efficacy of a videoconference for mental health care delivery in clinical settings (24), and the others studied the use of self-help Internet-based programs for the prevention or treatment of mental disorders, with or without support (25–27).

We also identified 15 naturalistic, acceptability, or feasibility studies. Three studies used Internet-based technologies to support mental health care delivery in clinical settings (28–30) and six studies that used guided self-help Internet-based programs to reach individuals with mental disorders outside clinical settings, promote treatment, or medication adherence, and provide ongoing encouragement and targeted psychosocial support (31–36). Likewise, we found four unguided Internet-based self-help programs for mental disorders and alcohol misuse (37–40) and two interventions oriented to provide basic mental health education to health-care workers (41, 42).

The main characteristics of each study are presented in **Tables 1**, **2**, and **3**, including population setting, aims of interventions, digital technology used, and main outcomes.

**Table 1:** Description of randomized controlled trial (RCT) studies of Internet-based interventions for mental health in Latin America.

Authors (year)/Country	Population Setting Age group *N*	Aim of the intervention	Study design Measurement time	Intervention	Outcome
Baldin et al. (2018) (21); Sanchez & Sanudo (2018) (22)/Brazil	Adults with alcohol abuse Night clubs ≥18 years old *N* = 1,057	Hazardous or harmful alcohol use reduction	Two-arm RCT Baseline and follow-up (3, 6, and 12 months after allocation	At baseline, participants were classified into two AUDIT score groups: “high risk” and “low risk.” In both groups, the intervention subgroup was exposed once to a normative feedback on alcohol consumption through a web-based screen, with additional information on the risks associated with the amount consumed, money spent on drinks, drinking, and driving, risk classification, and providing tips to reduce consumption.	In the “high-risk” group, an effect of the intervention was observed at 6 months, i.e., there was an estimated 13% reduction in the AUDIT score in favor of the intervention subgroup [odds ratio (OR) = 0.87; 95% confidence interval (CI): 0.76, 1.00]. After 12 months, no differences were found between the intervention and the control conditions in either risk group. There were significant reductions in both the AUDIT score and the prevalence of binge drinking over time in both the control and the intervention subgroups. In the “low-risk” group, participants in both arms had increased AUDIT scores.
Martínez et al. (2018) (23)/Chile	Adolescent patients with major depressive disorder Primary health care centers 13–19 years old *N* =143	Depressive symptomatology reduction	Two-arm RCT Baseline and follow-up (12 weeks)	Primary health-care teams received remote supervision by a psychiatrist through a shared electronic health record and a phone patient monitoring system. The intervention lasted 3 months.	Primary care clinicians were satisfied with the intervention, valuing its usefulness. However, there were no significant differences in depressive symptoms or health-related quality of life between groups. The adolescents in the intervention group were more satisfied with psychological assistance than those in the enhanced usual care group.
Pereira et al. (2015) (20)/Brazil	Primary school teachers Nine schools No age inclusion criteria *N* = 213	Improve knowledge, views, and attitudes about childhood mental disorders	Three-arm cluster RCT Pre- and postintervention (3 weeks)	Web-based program to educate primary school teachers about childhood mental disorders; consisting of tutorials, educational videos, online discussion forum, expert feedback and consultation with a psychiatrist, and written materials; compared with two control groups: one that received only text and video materials, and a waitlist control that received no intervention. The intervention lasted 3 weeks.	115 (54%) teachers completed follow-up. Teachers in the web-based program had greater gains in knowledge about mental disorders such as depression and conduct disorder (*p* < 0.05), and better understanding about mental disorders, such as less stigmatized views (*p* < 0.05), than did those in the text and video only and waitlist control groups. However, no differences were observed between the groups in attitudes about mental disorders.

**Table 2:** Description of pilot randomized controlled trial (RCT) studies of Internet-based interventions for mental health in Latin America.

Authors (year)/Country	Population Setting Age group *N*	Aim of the intervention	Study design Measurement time	Intervention	Outcome
Barrera et al. (2015) (27)/Argentina, Chile, Colombia, Mexico, Spain, and USA	Pregnant women Web-site ≥18 years old *N* = 852	New cases of postpartum depression reduction	Two-arm pilot RCT 3- and 6-month follow-up postpartum	Online intervention to prevent post-partum depression by encouraging women to create a healthy lifestyle for themselves and their newborn. The intervention lasted eight weekly sessions and included text, audio, and video materials and worksheets. The intervention was compared with an information only control group.	A small group [111 (13%)] completed follow-up, had complete data, and were included in the analysis. Levels of symptomatology did not differ significantly between groups, and the benefits of the intervention were higher for pregnant women reporting higher levels of depressive symptoms (*p* = 0.023).
Cárdenas López et al. (2014) (25)/Mexico	Adults with social anxiety No setting specified 19–60 years old *N* = 66	Fear of public speaking reduction	Three-arm pilot RCT Baseline and 3-month follow-up	Internet-based psychoeducation program for social anxiety based on cognitive behavioral techniques. It is composed by two active groups: self-guided and assisted by therapist in face to face. The telepsychology program consists of three modules dedicated to the evaluation, treatment and prevention of relapses. Within the exposure component, there were 10 scenarios consisting of videos of real audiences in public speaking situations.	43 participants (61% female) completed the intervention and follow-up. There was an improvement in measures of anxiety in both active groups, compared with the waitlist control (*p* < 0.05).
Hungerbuehler et al. (2016) (24)/Brazil	Outpatients Home-based 18–55 years old *N* = 107 (71% female)	Depressive symptomatology reduction	Two-arm pilot RCT 6- and 12-month follow-up	Telepsychiatry involving monthly online Skype videoconference consultations with psychoeducation, medication monitoring, and counseling with a psychiatrist and medication delivery to patients’ homes. The comparison group had monthly face-to-face consultations at the psychiatric hospital, and medications available at the clinic following the consultation.	85 (79%) participants completed 12-month follow-up. There were 489 video consultations and 461 face-to-face consultations; both groups had a reduction in depressive symptoms (*p* < 0.001) and an improvement in mental health status (*p* = 0.001). Clinical outcomes did not differ significantly between groups. Patients in both groups reported satisfaction with their treatment.
Tiburcio et al. (2018) (24)/Mexico	Individuals seeking treatment for substance abuse Addiction treatment centers ≥17 years old *N* = 74 (89% male)	Substance abuse and depressive symptomatology reduction	Three-arm pilot RCT Baseline, posttreatment, and follow-up at 1 month	PAADD is a web-based cognitive–behavioral intervention for the reduction in substance use and depression. The intervention incorporates the participation of a counselor who provides feedback and motivation through a messaging system. Completing the program requires approximately 8 weeks if used at least 1 h/week.	The results showed a reduction from baseline to follow-up in average days of use [*F*(1,28) = 29.70, *p* < 0.001], severity of use [*F*(2,28) = 143.66, *p* < 0.001], and depressive symptomatology [*F*(4) = 16.40, *p* < 0.001], independent of the type of treatment provided. The results suggest that this web-based intervention to reduce substance abuse is feasible, but the results showed high attrition rate.

**Table 3:** Description of naturalistic, acceptability, and feasibility studies of Internet-based interventions for mental health in Latin America.

Authors (year)/Country	Population Setting Age group *N*	Aim of the intervention	Study design Measurement time	Intervention	Outcome
Andrade et al. (2016) (38)/Brazil	General population Web-site No age inclusion criteria *N* = 929	Hazardous or harmful alcohol use reduction	Prospective naturalistic study (intrasubject pre–post study). Baseline, 6 weeks after baseline and follow-up (10 weeks after baseline)	“Beber menos” (Drink Less) is a web-based self-help cognitive–behavioral intervention for alcohol consumption reduction. This intervention includes alcohol use self-monitoring, goal setting with automated feedback, exercises to handle relapse and risky situations, weekly email reminders and progress reports, discussion forums, and an “ask a specialist” session. Users were invited to use the website for 6 weeks.	The results showed that 214 (29%) participants completed the 6-week follow-up. Among those completing the intervention, there was a reduction in alcohol consumption among harmful or hazardous users (63%) and suggestive substance misusers (65%) in comparison with baseline assessments (*p* = 0.02). Adherence to the program was low, but was higher among users at higher risk than among low-risk users.
Balsa et al. (2014) (39)/Uruguay	Students from 10 private schools Ninth and tenth grades *N* = 359	Substance misuse prevention	Descriptive naturalistic study Pre- and postintervention	COLOKT is a web-based substance misuse prevention intervention, which consists of educational materials, discussion forums mediated by a psychologist, and reminder SMS and emails. The intervention lasted 3 months.	The results showed that participation was low, 74 (21%) participants used the website only once. Predictors of website use included greater weekly Internet use, prior use of the Internet to search for health-associated topics, fewer extracurricular activities, and excessive alcohol consumption in the past month. Email and SMS reminders increased the interaction with the website.
Barrera-Valencia et al. (2017) (30)/Colombia	Male inmates Medium security prison ≥18 years old *N* = 106	Depression diagnosis and symptomatology reduction	Cost-effectiveness study Baseline and follow-up session	An asynchronous telepsychiatry (store-and-forward) intervention allowed primary care physicians to evaluate prisoners and send notes electronically to a consulting psychiatrist for diagnosis, treatment, and medication recommendations. This intervention of two sessions (assessment and follow-up session) was compared with a synchronous telepsychiatry model that involved videoconferencing consultation between the prisoner and a psychiatrist. The intervention lasted 6 months.	99 participants completed follow-up; both telepsychiatry models contributed to reduction in depressive symptoms among prisoners (*p* < 0.001). The asynchronous model showed a greater decrease in depressive symptoms than did the synchronous model (*p* = 0.01); the cost of the asynchronous model was significantly lower than that of the synchronous model (*p* < 0.001).
Campos et al. (2016) (40)/Colombia and Spain	Adults with flying phobia Setting not specified No age inclusion criteria *N* = 4	Flying phobia symptomatology reduction	Pilot acceptability study Baseline (treatment expectation) and after completion (treatment satisfaction)	NO-FEAR is an Internet-based self-help program that allows people with flying phobia to be exposed to images and sounds related to their phobic fears. The treatment protocol comprises: psychoeducation, exposure to scenarios composed by real sounds and images, and overlearning (the same exposure scenarios with greater difficulty).	Participants reported high expectations (*M* = 8.7; SD = 0.85) and satisfaction (*M* = 9.4; SD = 0.44) about the treatment on a scale from 1 to 10.
Cárdenas López et al. (2016) (34)/México	Victims and witnesses of assaults, kidnappings and criminal violence Psychological assistance center 18–65 years old *N* = 9	Posttraumatic stress disorder (PTSD) and acute stress disorder (ASD) symptomatology reduction	Nonrandomized open-label trial Pre- and posttreatment	90-min sessions conducted once a week by clinical psychologists. Between sessions 4–10, patients were exposed to 30–45 min Virtual Reality scenarios. The intervention lasted 10 weeks.	Treatment was successful in reducing PTSD and ASD symptoms from pre- to posttreatment. The posttreatment evaluation shows 30% of improvement in measures of stress, anxiety, and depression in both treatment groups. Although there was a significant effect of time (pre- vs. posttreatment, *p* < 0.001), there were no differences across groups (*p* > 0.05).
Carrasco (2016) (31)/Chile	Adolescents in treatment for depression Private and public health centers 12–18 years old Patients *N* = 15; Therapists *N* = 5	Depression symptomatology reduction (therapeutic resource)	Pilot feasibility and acceptability study Postintervention	Maya is an online adventure video game used for depression treatment among adolescents; narrative structure follows a hero’s journey. The scoring system provides cues about positive game behavior in the areas of: recognition and modification of negative cognitive bias; interpersonal skills and interpersonal problem solving; and behavioral activation and a healthy lifestyle. Information and resources are also available through the private online system. The study lasted 1 year.	The results showed that participants played the game for a mean 11.57 min (SD 3.42). Four participants played the game more than once; 13 participants completed acceptability ratings; 9 participants reported positive acceptability and considered the game beneficial; 4 participants did not find the game beneficial.
Espinosa et al. (2016) (32)/Chile	Patients discharged from depression treatment Private outpatient clinic 18–65 years old *N* = 35	Depressive symptomatology monitoring and relapse prevention	Pilot feasibility and acceptability study Postintervention	ASCENSO is an online program for relapse prevention after depression treatment. The program includes reminder emails and web-based modules for symptom monitoring, self-care recommendations, online counseling appointments with a psychologist, and information and resources. In case a patient reported severe impairment, the ASCENSO team contacted the patient to explore the need for further professional support. The study lasted 8 months.	The results showed that 23 (66%) participants actively used the program and were sent 330 reminders to monitor their depressive symptoms. Most participants reported that the program was beneficial and that the monitoring component was useful. Technical issues and limited time were cited as primary reasons for not using the program.
Flores et al. (2014) (29)/Mexico	University students with mild or moderate depression Universities 19–48 years old *N* = 8	Depressive symptomatology reduction	Pilot acceptability and intrasubject pre–post study Baseline, postintervention, and 6-month follow up	Internet-based CBT treatment with weekly sessions for 16 weeks. Communication was *via* chat, audio or videoconference.	The results showed a significant decrease between baseline and post-intervention in depressive (p = 0.012) and anxiety (p = 0.03) symptomatology. The gains remained at 6-month follow-up. The participants reported high satisfaction with the intervention.
Lara et al. (2014) (37)/México	Adults users who registered and entered the site two or more times in a 4-year period Website > 18 years old *N* = 17,318	Depressive symptomatology reduction	Descriptive naturalistic study Baseline, after completion of Module 3 (intermediate assessment), and after completion of Module 7 (final assessment)	ADEP is a free web-based psychoeducation, cognitive behavioral intervention that includes seven self-help modules with symptom assessment, feedback for users, vignettes, recorded messages, relaxation exercises, workbooks, blogs, and a discussion forum. These modules were free for participants to move at their own pace. Users were suggested to participate for 8 weeks.	The results showed high attrition rate: 5% of users completed all seven modules, 65% used the workbook, 61% used the discussion forum of which 16% added a post, and 67% contributed to the blogs. The participants made a good evaluation of the utility and usefulness of the modules. Because of the high attrition, there was no pre–post comparison of depressive symptomatology.
Menezes et al. (2019) (36)/Brazil, Peru	Patients in treatment for hypertension or diabetes Primary care health centers ≥21 years old *N* = 66	Depressive symptomatology reduction	Three pilot feasibility and acceptability studies (1 in São Paulo, Brazil, and 2 in Lima, Peru) Baseline and postintervention (6 weeks)	CONEMO is an app-based psychoeducational 6-week intervention assisted by a nurse for reducing depressive symptoms among individuals with diabetes or hypertension. CONEMO consists of 18 brief behavioral activation sessions, delivered over 6 weeks (3 sessions per week). As part of the behavioral activation program, CONEMO aims at increasing pleasant and health daily life activities, as well as providing information and health self-care messages.	The results showed that the intervention was feasible in both settings. There was a reduction in depressive symptoms as measured by PHQ-9 in all pilot studies. In total, 58% (38/66) of the participants reached treatment success rate (PHQ-9 < 10), with 62% (13/21) from São Paulo, 62% (13/21) from the first Lima pilot, and 50% (12/24) from the second Lima pilot study. The intervention was well received by participants in both settings.
Novaes et al. (2012) (41)/Brazil	Health-care professionals Family health-care centers No age inclusion criteria *N* = 1,422	Improve knowledge about mental health	Pre–post study 2-month follow-up	Tele-education program consisting of weekly web conference seminars and moderated discussion forums to provide education to family health-care teams about mental health.	The results showed 39 tele-education sessions were done during the 1-year period, and 384 (27%) health professionals responded to follow-up evaluations; nearly all respondents were satisfied with the program and thought that the seminars contributed to their professional development; two-thirds reported difficulties with video and audio connectivity.
Pereira et al. (2015b) (42)/Brazil	Health-care professionals working in primary care settings Website No age inclusion criteria *N* = 100	Improve knowledge about alcohol misuse misconceptions and management	Pre–post study 2-month follow-up	Online course to enhance health professionals’ knowledge about the clinical management of alcohol misuse; course consisted of nine instructor-led classes and web conferences, video exhibitions, text materials, and online chats and forums.	The results showed that only 33 of 100 enrolled participants completed the course. Among them, it was observed a significant improvement in knowledge about the clinical management of alcohol-related problems (*p* < 0.001) but no improvement in understanding about misconceptions and biases related to alcohol problems. Participants expressed satisfaction with the course.
Rojas et al. (2018) (28)/Chile	Patients in treatment for depression Community hospitals located in rural areas 18–70 years old *N* = 250	Depressive symptomatology reduction	Non-randomized two-arm open-label (blinded outcome assessor) trial; compared with usual care Baseline and follow-up (3 and 6 months after assignment)	A remote collaborative depression care intervention for patients living in rural areas through shared electronic health records (SEHR) between primary care teams and a specialized mental health team, and telephone monitoring of patients. The intervention lasted 3 months.	The intervention achieved higher user satisfaction [odds ratio (OR) 1.94, 95% CI 1.25–3.00] and better treatment adherence rates (OR 1.81, 95% CI 1.02–3.19) at 6 months compared to usual care. There were no statically significant differences in depressive symptoms between the intervention group and usual care, but a trend was observed in favor of the first one. Significant differences between groups in favor of the intervention group were observed at 3 months for mental health-related quality of life (beta 3.11, 95% CI 0.19–6.02).
Solorzano et al. (2010) (35)/Nicaragua, Guatemala, Costa Rica	Teens development organizations 10–20 years old *N* = 3,998	Health promotion	Survey questionnaire of feasibility (use) and acceptability (satisfaction) at the end of the calendar year.	TeenSmart is a web-based education tools and services for adolescent health promotion. It is integrated into existing organizational programs, curricula, and activities. The different tools of the program are provided in an interactive (requiring feedback) and/or noninteractive (information only) way.	Two-thirds of the teachers and other youth development organizational staff reported sufficient administrative support, ability to develop a leadership team, and an annual plan for integrating the TeenSmart tools into existing curricula and activities. More than 87% of teenagers reported that the website materials were easy to follow and understand, and 67% reported being completely satisfied with the virtual facilitator’s communications. The teens were less satisfied with the length of reading materials and recommended more dynamic material.
Vanegas et al. (2017) (33)/Colombia	Patients discharged from major depression treatment No setting specified 18–65 years old *N* = 15	Depression symptomatology monitoring and relapse prevention	Pilot feasibility and acceptability study PHQ-9 was applied once every 2 weeks	ASCENSO is an online program to support depression treatment and prevent relapse; the program includes reminder emails and web-based modules for symptom monitoring, self-care recommendations, online counseling appointments with a psychologist, and information and resources. In case a patient reported severe impairment, the ASCENSO team contacted the patient to explore the need for further professional support. The study lasted 8 weeks.	The results showed that 26.5% of those registered decided not to use ASCENSO. Twenty percent of the participants dropped out of the process after answering the first monitoring; 46.5% made partial use of the program; and 7% answered all the programmed monitoring. The results show a favorable opinion of participants.

### Results of Individual Sources of Evidence

#### Randomized Controlled Trials

In Brazil, a web-based educational program was designed to educate primary school teachers on the recognition and classroom management of children with mental health problems through interactive tutorials, educational videos, and an online discussion forum with mental health experts (20). Teachers receiving this web-based program had greater gains in knowledge about mental disorders such as depression and conduct disorder, and less stigmatized views on mental disorders, than did those trained with a text- and video-based program and those receiving no training. These results suggest that web-based interactive tools can be more effective than traditional educational tools in increasing knowledge and reducing stigma. However, in spite of increased knowledge, the impact on improving recognition and referral could not materialize.

In addition, in Brazil, a web-based intervention aimed at decreasing alcohol consumption and/or preventing alcohol abuse among nightclub patrons demonstrated significant reductions in reducing scores on the Alcohol Use Disorders Identification Test (AUDIT) and the prevalence of binge drinking over time in a “high-risk” group (21, 22). However, in a “low-risk” group, AUDIT scores increased in those receiving the intervention and a control group, and there were no differences in the prevalence of binge drinking across groups or compared with baseline levels.

In Chile, an online platform facilitated collaborative care by giving primary care providers remote access to a psychiatrist who supported the treatment of adolescent depression (23). The intervention proved to be feasible and well accepted by both patients and primary care clinicians. Satisfaction with psychological care, in both groups, was related to a significant change in depressive symptomatology at 12 weeks follow-up. However, at follow-up, the intervention did appear to have equivalent effectiveness to reduce depressive symptoms compared with enhanced usual care.

#### Pilot Randomized Controlled Trials

In Brazil, monthly telepsychiatry consultations through videoconferencing for the care of people with depression seem to achieve similar clinical outcomes as standard face-to-face treatment regarding mental health status, satisfaction with treatment, treatment adherence, or medication compliance (24). An interesting result is that participants in the videoconferencing group were able to establish an equivalent therapeutic relationship as those treated in person. However, all these results need to be taken with caution, as this was a feasibility study not statistically powered to test any of these differences.

In Mexico, two studies used guided self-help Internet-based programs. One study explored the feasibility of an Internet-based psychoeducation program for social anxiety (25). A module of the program incorporated cognitive–behavioral techniques such as cognitive restructuring and exposure to scenarios depicting real audiences in public-speaking situations through videos. The results supported the feasibility of this program and showed a tentative clinical improvement in measures of anxiety. A relevant aspect to consider is the difference in attrition rate across groups, with the group assisted by therapist showing the lowest (27%) while the group without treatment the highest (45%). In the self-applied group, it seems that some of the attrition can be explained by problems with accessing Internet and achieve the necessary immersion in the exposure scenarios.

Another study developed a web-based cognitive–behavioral intervention (CBT) for the reduction in substance use and depressive symptomatology (PAADD) and compared it with two other CBT brief interventions: an in-person session with an addiction therapist combined with ASSIST Self-help Strategy Guide and the treatment ordinarily offered at participating treatment centers (26). The results suggest that CBT strategies were successfully translated to web format in the PAADD, with possibilities similar to those of brief interventions carried out in person and with printed materials. This intervention appears to be feasible and shows a potential reduction of participants’ depressive symptomatology, although it was not demonstrated to be more effective than the other interventions. Regrettably, the intervention presented problems associated with the high dropout rate.

Finally, a multisite study implemented an unguided Internet-based self-help program for the prevention of postpartum depression in different Latin American countries and the USA (27). Although this study showed that online self-help programs could be delivered and promote mental health among individuals in low-resource settings, it showed high dropout rates and failed to suggest that results could potentially be beneficial. In this respect, it seemed that benefits of receiving the intervention were greater for pregnant women reporting high (vs. low) levels of prenatal depression symptoms.

### Naturalistic, Acceptability, or Feasibility Studies

#### Telepsychiatry/Psychology

In Chile, an online platform facilitated collaborative care by giving primary care providers remote access to a psychiatrist (28). The intervention was feasible and achieved higher user satisfaction. This program also shows better treatment adherence rates at 6 months compared to usual care, suggesting that technology-assisted interventions may help rural primary care teams in the management of depressive patients. While this is not a randomized clinical trial, the potential effectiveness of this intervention seems comparable to the usual care delivered by a national depression treatment program.

In Mexico, a 16-session telepsychology treatment for depression (via chat, audio, or videoconference) seems potentially beneficial in decreasing depressive symptomatology, with gains remaining at 6-month follow-up (29). However, the quality of the study and the small sample size (*n* = 8) do not allow many conclusions to be drawn from this intervention.

In Colombia, a telepsychiatry intervention for depression management among prison inmates was compared with asynchronous teleconsulting with a psychiatrist through a web-based platform where the primary care professional present clinical information to the psychiatrist (30). Both interventions showed depression symptomatology reduction, but the asynchronous teleconsulting intervention proved to be less costly than the telepsychiatry intervention.

#### Guided Self-Help Internet-Based Programs

Two pilot studies from Chile showed acceptable online depression treatment programs, including an online video game for supporting depression treatment among adolescents (31) and an online program for symptom monitoring in patients receiving treatment for major depression (32). The online adventure video game is a psychotherapeutic tool for female adolescents in psychotherapy with mild to moderate depression (31). In the game, players follow the story of a female adolescent, who gets involved in interpersonal situations that require psychosocial reasoning. The majority of participants (patients and therapist) valued the game and considered that they could obtain mental health-related benefits from playing it. However, the time of use of the game was very low. Therapists also suggest that it was possible in postgame sessions to relate elements of the game to aspects of the patients’ real lives. Nevertheless, the design of the study and the small number of participants make it impossible to draw conclusions regarding the effectiveness of the game.

Espinosa et al. (32) studied the feasibility and acceptability of the Chilean version of the Supportive Monitoring and Disease Management over the Internet program (SUMMIT) (43), which was called in Spanish ASCENSO. This program aims to monitor and support patients after being discharged from depression treatment. Most of the participants displayed a good level of acceptance and generally regarded the program as a source of support and as beneficial; however, only half of them actively used the program and/or the online chat available. In Colombia, where the ASCENSO program was replicated, the results showed also a favorable acceptability from the participants, but there were important problems with usability and attrition (33). One of the main limitations of its implementation was associated with the difficulty of involving mental health institutions in the use of the program.

In Mexico, a study aimed to explore the feasibility of a program aimed to people who were victims of assaults, kidnappings, and criminal violence and suffered from posttraumatic stress disorder (PTSD) and/or acute stress disorder (ASD) (34). Participants were exposed to Virtual Reality scenarios and asked to talk about the traumatic event in the first person. The results showed that treatment was well received and potentially useful in reducing PTSD and ASD symptoms from pre- to posttreatment. Regarding the satisfaction of participants with the intervention, it is interesting to note that they did not manifest a preference between virtual reality exposure techniques and traditional exposure therapy (*in vivo* or imagined).

A study conducted in Nicaragua, Guatemala, and Costa Rica developed an Internet-based education program to promote adolescent health, integrating the program in existing organizational curricula and activities (35). This program was delivered in an interactive (requiring feedback) and/or noninteractive fashion (information only). The program proved to be feasible and provided a confidential way for youth and family development organizations to gather information about teenagers’ health behaviors and needs. Nevertheless, access and integration of materials and methods into existing curricula was a major problem, possibly associated with limited access to computers and the Internet in general.

A study conducted in Brazil and Peru developed a psychoeducational technological platform delivered *via* mobile phones to patients and assisted by a nurse holding a tablet dashboard to monitor progress in the reduction of depressive symptoms among individuals with comorbid diabetes or hypertension (36). The platform (CONEMO) aims at increasing daily life activities and motivation, as well as providing further information and health self-care messages. This tool demonstrated to be feasible in both settings, where three pilot studies were conducted, and participants were satisfied with the intervention. The samples were small to test the efficacy of the intervention. However, there was a trend in all pilot studies for a reduction in depressive symptoms over time. A fully powered RCT is in progress.

#### Unguided Internet-Based Self-Help Programs

In Mexico, a study collected data on individuals who entered a Mexican open access free web-based psycho-education and CBT intervention for depression (37). Data showed that adherence dropped considerably as individuals progressed through the intervention modules. However, all modules were rated very high for helpfulness/usefulness.

In Brazil, a program seemed beneficial for reducing alcohol consumption among harmful users and those with probable dependence, but program adherence was low (38). In Uruguay, a web-based substance misuse prevention intervention was implemented in 10 private schools (39). This study shows that sending participants periodic reminders *via* e-mail and SMS text messages has a positive impact in engagement with the program. Despite the adolescent-friendly design and the provision of social networking tools and interactive dynamics, low participation and high attrition were observed during the intervention. In Colombia, participants with a flying phobia that went through a program for treating their phobia with psychoeducation and exposure techniques reported high satisfaction with the intervention (40).

#### Internet-Based Programs for Education and Training of Health/Educational Workers

In Brazil, a tele-education program consisted of weekly web conference seminars and moderated discussion forums to provide education to family health-care teams about mental health (41). While participants felt that the seminars contributed to their professional development, the implementation of the tele-education program faced significant obstacles. Two major challenges were associated with Internet connectivity and the insertion of new technologies into the daily lives of health professionals, especially physicians.

In addition, in Brazil, an online course was oriented to enhance primary care professionals’ knowledge about the clinical management of alcohol misuse (42). In this study, health-care workers expressed satisfaction with the course, and a significant improvement in their knowledge about the clinical management of alcohol-related problems was observed. This indicates that e-learning is a useful medium for teaching mental health issues. However, this intervention also showed a number of difficulties. First, there was no reduction in stigma and prejudice related to alcohol problems. Second, the comparison between pre- and postcourse scores suggests that general knowledge about alcohol addiction did not improve over time. Finally, the lack of a control group did not allow the performance of e-learning to be compared with traditional face-to-face teaching.

### Risk of Bias Across Studies

**Table 4** presents a synthesis of the risk of bias assessment of the three included RCTs.

**Table 4:** Risk of bias summary.

	Random sequence generation (selection bias)	Allocation concealment (selection bias)	Blinding or participants and personnel (performance bias)	Blinding of outcome assessment (detection bias)	Incomplete outcome data addressed (attrition bias)	Selective reporting (reporting bias)
Pereira et al. (20)	✓	✓	**×**	**×**	**?**	✓
Balding et al. (21), Sanchez and Sanudo (22)	✓	✓	**×**	**?**	✓	✓
Martinez et al. (23)	✓	✓	**×**	✓	✓	✓

Low risk of bias: ✓ | High risk of bias: **×** | Unclear risk of bias: **?**

The first included RCT (20) describes the use of random sequences to generate the allocation and adequate concealment of allocation prior to assignment. In this type of intervention, it does not seem feasible to blind participants and personnel. Thus, the risk of bias for this item is described as “high.” The publication does not describe the measures used to blind outcome assessors to treatment allocation, so a high risk of detection bias can be inferred. This study describes the outcome data and the number of participants in each group. While this study included replacing missing data through imputation methods, there is a lack of information regarding the specific reasons for attrition. Thus, we assess the risk of attrition bias as “unclear.” The publication includes all prespecified results.

The second included RCT (21, 22) describes the use of random sequences to generate the allocation and adequate concealment of allocation prior to assignment. Participants were probably aware of allocation when they received feedback on their level of risk, so performance bias was identified during the conduct of the study. The outcomes were evaluated through self-report scales, eliminating observer bias. However, lack of information made it difficult to assess the blinding of outcome assessment, resulting in an “unclear” judgment on the risk of detection bias. This study describes the outcome data, reporting the reasons for attrition and exclusions, as well as the number of participants in each group. The publication includes all prespecified results.

The third RCT identified (23) describes that randomization was performed using computer generated random numbers with adequate allocation concealment. However, the participants knew the allocation. A trained consultant, who was blinded to the treatment allocation, assessed patient baseline data and outcomes at 12 weeks of follow-up. The study reports the reasons for attrition and exclusions, as well as the number of participants in each group. The publication includes all the prespecified results.

### Synthesis

Overall, the studies identified in this review are heterogeneous in terms of participants (e.g., adolescents, adults, patients, professionals), contexts (rural and urban, clinical and community settings), mental problems addressed (depression, anxiety, alcohol and substance misuse), and methods used to deliver the interventions (e.g., teleconsulting, online self-help programs, education and training of health/education workers, psychoeducation).

Within the 22 studies identified, only 3 studies were RCTs, 4 were pilot RCTs, and 15 were naturalistic, acceptability, or feasibility studies. Most of the feasibility and pilot studies showed reasonably good acceptability for a wide range of strategies but difficulties to engage and retain participants for long enough or adhering to established protocols. We found no large-scale effectiveness studies and no cost-effectiveness study. The methodological quality of RCTs studies identified was reasonably good but showed disparate results, and there are challenges to face. Overall, better results are seen in the short term (postintervention or after 3 months of follow-up), but most studies do not explore outcomes for long enough (follow-up after 6 or 12 months).

## Discussion

Internet-based interventions for mental disorders have already shown their potential benefits in high-income countries. The use of the Internet and digital technologies may improve access, enhance the flexibility of conventional treatments, facilitate the monitoring of treatment progress and fidelity with which interventions are delivered, and may improve the integration of different levels of care, obtaining results comparable to face-to-face care (11, 14, 44). However, much less is known about the feasibility and potential benefits of these interventions in LMIC. Although the penetration of technology and its application to health has been swift in all countries regardless of their level of development (12), there are many issues that remain to be resolved if these technologies want to be disseminated at scale. Among these, the relative lack of studies showing effectiveness of Internet-based interventions for mental disorders in LMICs (15, 16). Some questions still remain about the effectiveness of these interventions in settings with constrained resources.

In this scoping review, we identified 22 primary studies addressing Internet-based interventions for mental disorders across Latin American countries published until April 2019. Our findings indicate that there is a growing number of studies testing the feasibility and acceptability of Internet-based interventions for prevention, treatment, education, or facilitating self-management of mental disorders across various settings. However, very few tested the effectiveness of these interventions through RCT designs. This is not an issue just affecting Latin America but the field in general (12).

The studies identified in this review are heterogeneous, demonstrating the flexibility of Internet-based interventions to adapt to different population, contexts, and formats. Many of the studies have prioritized the use of online technologies to assist vulnerable groups in low-resource settings without considering the challenges involved in their use and in future implementation. As a reviewed study shows (34), in some regions of Latin America, especially Central American countries, there is still limited access to computers and connectivity with the Internet, let alone a chronic shortage of supervisory human resources, all of which can become major barriers to the successful implementation of Internet-based interventions. Similarly, a study conducted in Brazil showed significant obstacles to implementing at larger scale a tele-education program not only because of low connectivity but also because of the challenge of introducing new technologies in the daily work of busy practitioners in public health contexts (41). Future studies should pay more attention to these potential challenges when it comes to bring innovation to scale; otherwise, the solutions developed and tested at high cost will never be scaled up.

Most studies had methodological limitations such as poorly defined samples, unclear comparison groups, and lack of randomization methods, and interventions of short duration. One of the most important problems identified is that most of these studies compared results across groups in terms of efficacy, even though none of them was statistically powered to estimate differences across groups. Notwithstanding this, most studies showed a trend suggesting increased benefits when compared to control groups, whenever these had been included in the study design. Caution must be exercised when presenting results of feasibility or pilot studies. A common finding in mental health interventions is that early gains tend to fade away unless there is continuous intervention over time; most studies included were short-term. There is a need for studies that explore outcomes beyond six months; however, it is acknowledged that funding for longer lasting feasibility studies is hard to get.

Some interventions were unguided, but most of them included guidance or support that varied from low-intensity support (feedback and motivation) to using the technology as a means to deliver synchronous assessments, therapy, or supervision (telepsychiatry, telepsychology, or teleconsultation through videoconference). Most interventions adopted evidence-based techniques of traditional face-to-face treatments, which indicate that there is still ample scope for innovation in Internet-supported techniques. There is growing awareness that guided intervention tends to achieve better results, but there is still not enough knowledge in terms of the most cost-effective options to provide this guidance and the options to innovate in this respect are endless. In the case of Latin America, it is possible to explore various options to adopt the “blended approach,” since there is still a large supply of reasonably trained health workers who can assist in this process. However, resources are finite, and there are competing duties that need to be accounted for when planning for human guidance and support. Guided Internet-therapies have the potential to improve effectiveness and reach of psychological support and treatment for mental disorders in developing countries (16), without disproportionately increasing costs for health services.

The lack of specialized human resources is a critical issue in delivering mental health care in LMIC (5, 6). The training of nonspecialist professionals (primary health-care providers, or community health-care workers) with tools to diagnose and detect mental disorders is an important area of development that aims to reduce the treatment gap. As some reviewed studies show, the use of the Internet provides an option to overcome this problem, by delivering online training on mental health for primary healthcare professionals and/or providing remote communication between them and mental health specialists. This can be fundamental in task-shifting strategies from specialist medical providers to less well-trained personnel. However, as this review shows, further research is needed in this area, especially on how to change attitudes towards mental disorders.

Internet-based interventions for mental health identified in this review go beyond clinical settings. A few identified studies targeting school populations, both students and teachers. The results of a web-based educational program included in this review (20) suggest that web-based interactive tools can be more effective than traditional educational tools in increasing knowledge and reducing stigma. This is an interesting result, considering that stigma and misconceptions related to mental health are commonly seen in the educational sector (45). Schools have become relevant spaces for mental health promotion, indicated prevention and early detection of mental health problems. A reduction in stigma related to mental health problems might improve the referral process from the educational sector. To improve teachers’ knowledge on mental health, online training appears as a promising strategy, with benefits like flexible usage not constrained by time and place and easy dissemination through national territories.

High attrition in Internet-based interventions where there is none or low-intensity coach/therapist support is a problem in Latin America as well as in developed countries (11, 15). Future studies should explore in detail how important is the human support in Latin America for increasing engagement and adherence as well as improving outcomes. In addition, other strategies for increasing adherence, like the inclusion of persuasive systems or user-centered designs (46), need to be evaluated. Other aspect less addressed by the included studies is how the integration of these interventions to the existing health networks in which the patient is already inserted could increase their acceptability, adherence, and efficacy.

Overall, this study shows that Internet-based interventions for the prevention and treatment of mental disorders are at an early stage of development in Latin America. The accumulating evidence shows promising results but with important challenges that are not different to those found elsewhere in the world (11–16). There is an urgent need to agree on evaluation methodologies and frameworks to assess the growing number of emerging interventions in this field (13). It is also necessary to produce methodological developments in large-scale effectiveness studies, as well as cost effectiveness and implementation studies, especially in primary care services. Future studies should also place greater emphasis on comparing online interventions with traditional face-to-face interventions, either alone or in combination. Progress in these areas is a necessary condition for scaling up these interventions and obtaining funding from health insurers.

### Strengths and Limitations

As far as we know, this is the first review of Internet-based interventions for mental disorders that specifically addresses developments in this area in Latin America. However, our scoping review has some limitations. An important limitation is related to the small number of studies and publications found, in particular, the scarcity of randomized clinical trials. There are many feasibility studies of commercial applications and products that are not published anywhere. Likewise, pilot studies for a range of mental disorders have been carried out, and there are many which are developed for commercial purposes, but there is no reporting of its quality or outcomes. There is little consistency in the methodologies used in feasibility and pilot studies, which interferes further when comparing studies. Another important limitation is that four authors of this article (RA, GR, VM, and PM) are also responsible for some of the studies reviewed, which can be a source of many biases in the interpretation of the results. Nevertheless, the risk of bias assessment of RCT studies was carried out by ÁJ-M and PF, who did not participate in any of the studies reviewed. Besides, it shows that there is a group of local researchers in the field that is eager to learn from the experiences of others.

## Conclusion

Internet-based interventions of mental disorders are growing rapidly in countries at all levels of development. The aim of this scoping review was to systematically map the existing evidence on use of Internet-based interventions for prevention, treatment, education, or facilitating self-management of mental disorders in Latin America, as well as to identify existing gaps in the literature. The results show that there are a growing number of studies testing the feasibility and acceptability of interventions, but there are few studies on effectiveness and cost effectiveness. Furthermore, there are few studies comparing the efficacy of Internet-based interventions with traditional face-to-face interventions. The relative lack of evidence conspires against efforts to disseminate and scale up digital interventions in the region. These results lead us to advocate for increasing the number of studies and, more importantly, improving the quality of e-mental health research in Latin America to produce better evidence to guide mental health policies.

## Data Availability

All datasets generated for this study are included in the manuscript/**Supplementary files**.

## Author Contributions

ÁJ-M, RA, GR, VM, and PM contributed conception and design of the study; PM and ÁJ-M organized the database; ÁJ-M, PF, VM, and RA wrote the first draft of the manuscript. All authors contributed to manuscript revision, read, and approved the submitted version.

## Funding

This study was supported by the Millennium Science Initiative of the Ministry of Economy, Development and Tourism, grant “Millennium Nucleus to Improve the Mental Health of Adolescents and Youths, Imhay,” grant “Millennium Nucleus in Social Development, Desoc,” and the Fund for Innovation and Competitiveness (FIC) of the Chilean Ministry of Economy, Development and Tourism, through the Millennium Science Initiative, Grant No. IS130005. PF received funding from CONICYT PFCHA/DOCTORADO NACIONAL/2019-21190745.

## Conflict of Interest Statement

The authors declare that the research was conducted in the absence of any commercial or financial relationships that could be construed as a potential conflict of interest.
