# Supplementary material for: Internet-Based Interventions for the Prevention and Treatment of Mental Disorders in Latin America: A Scoping Review
Source: Front Psychiatry. 2019 Sep 13;10:664. doi: 10.3389/fpsyt.2019.00664 (PMC6753742; doi:10.3389/fpsyt.2019.00664)
Supplement: Supplementary file 1 [file DataSheet_1.docx]

Supplementary Material

# Search strategies

***Pubmed***

(Belize[Title/Abstract] OR Costa Rica[Title/Abstract] OR El Salvador[Title/Abstract] OR Guatemala[Title/Abstract] OR Honduras[Title/Abstract] OR Nicaragua[Title/Abstract] OR Panama[Title/Abstract] OR Mexico[Title/Abstract] OR Argentina[Title/Abstract] OR Bolivia[Title/Abstract] OR Brazil[Title/Abstract] OR Chile[Title/Abstract] OR Colombia[Title/Abstract] OR Ecuador[Title/Abstract] OR French Guiana[Title/Abstract] OR Guyana[Title/Abstract] OR Paraguay[Title/Abstract] OR Peru[Title/Abstract] OR Suriname[Title/Abstract] OR Uruguay[Title/Abstract] OR Venezuela[Title/Abstract] OR Central America[Mesh] OR South America[Mesh]) AND (Psychiatry and Psychology Category[Mesh] OR Psychiatry[Mesh] OR Psychology[Mesh] OR Anxiety[Title/Abstract] OR Bipolar[Title/Abstract] OR Mood[Title/Abstract] OR Depression[Title/Abstract] OR Schizophrenia[Title/Abstract] OR Psychosis[Title/Abstract] OR Alcohol[Title/Abstract] OR Substance Abuse[Title/Abstract] OR Drug Use[Title/Abstract]) AND (Acceptability[Title/Abstract] OR Effectiveness[Title/Abstract] OR Efficacy[Title/Abstract] OR Feasibility[Title/Abstract]) AND ("Information Science"[Mesh] OR Internet[Title/Abstract] OR Computer[Title/Abstract] OR Technolog*[Title/Abstract] OR Digital[Title/Abstract] OR Electronic[Title/Abstract])

***Embase***

(‘Belize’:ab,ti OR ‘Costa Rica’:ab,ti OR ‘El Salvador’:ab,ti OR ‘Guatemala’:ab,ti OR ‘Honduras’:ab,ti OR ‘Nicaragua’:ab,ti OR ‘Panama’:ab,ti OR ‘Mexico’:ab,ti OR ‘Argentina’:ab,ti OR ‘Bolivia’:ab,ti OR ‘Brazil’:ab,ti OR ‘Chile’:ab,ti OR ‘Colombia’:ab,ti OR ‘Ecuador’:ab,ti OR ‘French Guiana’:ab,ti OR ‘Guyana’:ab,ti OR ‘Paraguay’:ab,ti OR ‘Peru’:ab,ti OR ‘Suriname’:ab,ti OR ‘Uruguay’:ab,ti OR ‘Venezuela’:ab,ti OR ‘South America’/exp OR (‘Central America’/exp NOT ‘Caribbean’/exp)) AND (‘mental disease’/exp OR ‘mental health’/exp OR ‘psychiatry’/exp OR ‘psychology’:ab,ti OR ‘anxiety’:ab,ti OR ‘bipolar’:ab,ti OR ‘mood’/exp OR ‘depression’:ab,ti OR ‘schizophrenia’:ab,ti OR ‘psychosis’:ab,ti OR ‘alcohol’:ab,ti OR ‘substance abuse’:ab,ti OR ‘drug use’:ab,ti) AND (‘acceptability’:ab,ti OR ‘effectiveness’:ab,ti OR ‘efficacy’:ab,ti OR ‘feasibility’:ab,ti) AND (‘information science’/exp OR ‘internet’:ab,ti OR ‘computer’:ab,ti OR ‘technolog*’:ab,ti OR ‘digital’:ab,ti OR ‘electronic’:ab,ti)

***Cumulative Index to Nursing and Allied Health Literature (CINAHL)***

((TI Belize) OR (AB Belize) OR (TI Costa Rica) OR (AB Costa Rica) OR (TI El Salvador) OR (AB El Salvador) OR (TI Guatemala) OR (AB Guatemala) OR (TI Honduras) OR (AB Honduras) OR (TI Nicaragua) OR (AB Nicaragua) OR (TI Panama) OR (AB Panama) OR (TI Mexico) OR (AB Mexico) OR (TI Argentina) OR (AB Argentina) OR (TI Bolivia) OR (AB Bolivia) OR (TI Brazil) OR (AB Brazil) OR (TI Chile) OR (AB Chile) OR (TI Colombia) OR (AB Colombia) OR (TI Ecuador) OR (AB Ecuador) OR (TI French Guiana) OR (AB French Guiana) OR (TI Guyana) OR (AB Guyana) OR (TI Paraguay) OR (AB Paraguay) OR (TI Peru) OR (AB Peru) OR (TI Suriname) OR (AB Suriname) OR (TI Uruguay) OR (AB Uruguay) OR (TI Venezuela) OR (AB Venezuela) OR (MH “Central America+”) OR (MH “South America+”)) AND ((MH “Behavior and Behavior Mechanisms+”) OR (MH “Psychological Processes and Principles+”) OR (MH “Behavioral and Mental Disorders+”) OR (MH “Psychiatry+”) OR (MH “Psychology+”) OR (TI Anxiety) OR (AB Anxiety) OR (TI Bipolar) OR (AB Bipolar) OR (TI Mood) OR (AB Mood) OR (TI Depression) OR (AB Depression) OR (TI Schizophrenia) OR (AB Schizophrenia) OR (TI Psychosis) OR (AB Psychosis) OR (TI Alcohol) OR (AB Alcohol) OR (TI Substance Abuse) OR (AB Substance Abuse) OR (TI Drug Use) OR (AB Drug Use)) AND ((TI Acceptability) OR (AB Acceptability) OR (TI Effectiveness) OR (AB Effectiveness) OR (TI Efficacy) OR (AB Efficacy) OR (TI Feasibility) OR (AB Feasibility)) AND ((MH "Information Science+") OR (TI Internet) OR (AB Internet) OR (TI Computer) OR (AB Computer) OR (TI Technolog*) OR (AB Technolog*) OR TI (Digital) OR (AB Digital) OR (TI Electronic) OR (AB Electronic))

***Web of Science Core Collection (Web of Science)***

TS=(Belize OR Costa Rica OR El Salvador OR Guatemala OR Honduras OR Nicaragua OR Panama OR Mexico OR Argentina OR Bolivia OR Brazil OR Chile OR Colombia OR Ecuador OR French Guiana OR Guyana OR Paraguay OR Peru OR Suriname OR Uruguay OR Venezuela OR Central America OR South America) AND TS=(Mental Health OR Mental Disorders OR Mental Disease OR Psychological Processes OR Behavioral and Mental Disorders OR Psychiatry OR Psychology OR Anxiety OR Bipolar OR Mood OR Depression OR Schizophrenia OR Psychosis OR Alcohol OR Substance Abuse OR Drug Use) AND TS=(Acceptability OR Effectiveness OR Efficacy OR Feasibility) AND TS=(Information Science OR Internet OR Computer OR Technolog* OR Digital OR Electronic)

***SciELO Citation Index (Web of Science)***

TS=(Belize OR Costa Rica OR El Salvador OR Guatemala OR Honduras OR Nicaragua OR Panama OR Mexico OR Argentina OR Bolivia OR Brazil OR Chile OR Colombia OR Ecuador OR French Guiana OR Guyana OR Paraguay OR Peru OR Suriname OR Uruguay OR Venezuela OR Central America OR South America) AND TS=(Mental Health OR Mental Disorders OR Mental Disease OR Psychological Processes OR Behavioral and Mental Disorders OR Psychiatry OR Psychology OR Anxiety OR Bipolar OR Mood OR Depression OR Schizophrenia OR Psychosis OR Alcohol OR Substance Abuse OR Drug Use) AND TS=(Acceptability OR Effectiveness OR Efficacy OR Feasibility) AND TS=(Information Science OR Internet OR Computer OR Technolog* OR Digital OR Electronic)

***Cochrane Controlled Register of Trials (CENTRAL)***

(Belize:ti,ab OR "Costa Rica":ti,ab OR "El Salvador":ti,ab OR Guatemala:ti,ab OR Honduras:ti,ab OR Nicaragua:ti,ab OR Panama:ti,ab OR Mexico:ti,ab OR Argentina:ti,ab OR Bolivia:ti,ab OR Brazil:ti,ab OR Chile:ti,ab OR Colombia:ti,ab OR Ecuador:ti,ab OR "French Guiana":ti,ab OR Guyana:ti,ab OR Paraguay:ti,ab OR Peru:ti,ab OR Suriname:ti,ab OR Uruguay:ti,ab OR Venezuela:ti,ab OR [mh “Central America”] OR [mh “South America”]) AND ([mh “Psychiatry and Psychology Category”] OR [mh “Psychiatry”] OR [mh “Psychology”] OR Anxiety:ti,ab OR Bipolar:ti,ab OR Mood:ti,ab OR Depression:ti,ab OR Schizophrenia:ti,ab OR Psychosis:ti,ab OR Alcohol:ti,ab OR "Substance Abuse":ti,ab OR "Drug Use":ti,ab) AND (Acceptability:ti,ab OR Effectiveness:ti,ab OR Efficacy:ti,ab OR Feasibility:ti,ab) AND ([mh "Information Science"] OR Internet:ti,ab OR Computer:ti,ab OR Technolog*:ti,ab OR Digital:ti,ab OR Electronic:ti,ab)

**
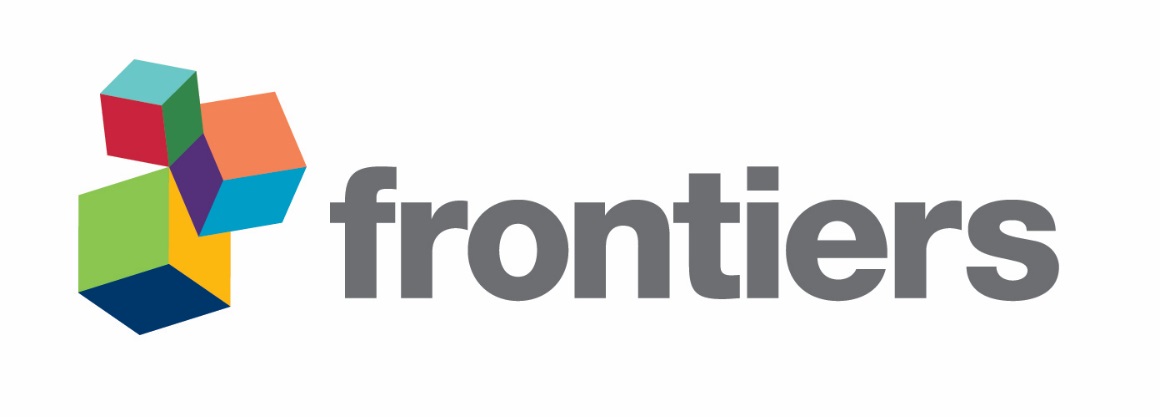
**
